# Burden and Psychological Distress of Intensive Care Unit Caregivers of Traumatic Brain Injury Patients

**DOI:** 10.5005/jp-journals-10071-23164

**Published:** 2019-05

**Authors:** Thiruchengodu Raju Kanmani, Ramappa M Thimmappur, Raju Birudu, Krishna Reddy N, Prabhu Raj

**Affiliations:** 1,2,4 Department of Psychiatric Social Work, National Institute of Mental Health and Neurosciences, Bengaluru, Karnataka, India; 3 Department of Psychiatry, National Institute of Mental Health and Neurosciences, Bengaluru, Karnataka, India; 5 Department of Neurosurgery, National Institute of Mental Health and Neurosciences, Bengaluru, Karnataka, India

**Keywords:** Family burden, ICU caregivers, Psychological distress, Traumatic brain injury

## Abstract

**Aim:**

Admission to Intensive Care Unit (ICU) is a stressful event and unforeseen crisis for the caregivers. Burden and psychosocial distress among caregivers in the ICU were unexplored. Therefore, the current study was aimed to assess the caregivers’ burden and psychological distress among caregivers of traumatic brain injury (TBI) patients at emergency ICU during hospitalization.

**Materials and Methods:**

A total of 60 caregivers recruited by using purposive sampling method with descriptive research design. Consent was obtained. Interview schedule of family for depression, anxiety, stress scale (DAS-21) were administered. The data were analyzed by using SPSS. Descriptive statistics and independent burden t-test were used.

**Results:**

Results revealed that male caregivers (75%) and female caregivers (25%) took part in the study. Caregivers' mean age was found to be 35.22±11.29 years. Most of the TBI survivors admitted in ICU had severe injury (8.30±3.63). Mean scores showed that caregivers had experienced financial burden (6.28±2.36), severe depression (12.15±4.84), and a moderate level of anxiety (12.85±5.20). Independent t-test showed significant difference in caregiving burden between male and female caregivers at ICU (Male = 18.43±4.83; Female = 14.29±4.83; t = 2.16; *p* <0.035). Overall, caregivers experienced higher family burden and severe psychological distress at ICU.

**Conclusion:**

There is an immediate need to assess psychological distress and family burden of caregivers at ICU and provide timely psychosocial intervention.

**How to cite this article:**

Kanmani TR, Thimmappur RM, Birudu R, Reddy KN, Raj P. Burden and Psychological Distress of Intensive Care Unit Caregivers of Traumatic Brain Injury Patients. Indian J Crit Care Med 2019;23(5):220-223.

## INTRODUCTION

Admission to intensive care unit (ICU) is a stressful event and unforeseen crisis perceived by patients as well as family members.^1^ Patients who are acutely ill require multiple and complex interventions. Most of the time, patients were unable to take care of themselves and depend on the caregivers.^2^ Severe traumatic brain injury (TBI) patients require ICU care for physical, functional, cognitive, and psychosocial support depending on the severity of the injury.^3^

Prognosis in TBI is not always predictable and depends on the severity of the injury. Traumatic brain injury is not only affecting the patient but it also affects the family members during the stay of patient in ICU.^4^ Caregiving during the hospital stay increases the responsibility of the caregiver in providing patients' daily care, treatment, and rehabilitation which surges the stress and burden.^5,6^ Caregivers assume caregiving is an important and necessary role during the ICU stay for their loved ones. They do act as surrogate decision makers and play a supportive role in fulfilling the hospital formalities and procedures on behalf of the patient.^2,7^ It is accepted that caregivers are an integral part of ICU care. Thus, incorporating the family centred approach in critical care is emphasized.^8,9^

Studies highlighted that caregivers experience high level of stress, anxiety, and depression within 3 to 5 days during ICU admission.^10^ In addition, changes in the role functions, family system, and family integrity are under risk.^11^ Research studies reported that financial crisis and accessing social support is difficult for caregivers during emergency time.^12^ It is also found that caregiver's unpreparedness to face crisis, unfamiliar hospital environment, lack of knowledge, timely communication, apprehensions regarding the prognosis of the patient's condition, and financial burden is associated with psychological distress. A study also identified that caregivers need varies based on the demographic characters like gender, religion, and ICU experience.^11^

Few Indian studies have explored on caregivers’ perceptions and their satisfaction on ICU services. Results showed that caregivers have poor understanding about ICU, higher satisfaction on ICU care received, and disappointment with ICU visiting hours. Further, caregiver concerns like feeling of loss, uncertainty, stress, and burden are not explored.^7,^^13–16^ A systematic review reported that psychosocial, behavioral, and mental health issues in the area of TBI are less investigated.^17^ Therefore, the current study was aimed to assess the family burden and psychological distress among TBI caregivers at emergency ICU.

## MATERIALS AND METHODS

The study adopted a descriptive research design, data were collected between July 2016 and January 2017 at Intensive Care Unit (ICU) in the surgical ICU in Emergency and Trauma Care centre in the tertiary hospital. Total number (n) of 60 respondents were recruited purposively with inclusion and exclusion criteria. Consented primary caregivers who were providing care to TBI patients at ICU for more than one week on regular basis and adults aged between 20 years and 60 years were recruited. Caregivers who were providing care to other neurological and neurosurgical patients were excluded. Sixty-four primary caregivers who met the inclusion criteria were contacted, of which four caregivers did not given consent to participate in the study, and thus excluded from the study. The data was collected in face to face interview by administering the interview schedule of family burden and depression, anxiety and stress scale (DAS-21).

To assess the caregiver burden, interview schedule of family burden was used.^18^ The scale has six domains, which measure the financial burden, disruption of daily routine work, disruption in family leisure, disruption of family interaction, the effect of physical health, and mental health. Each item was measured on 3 point scale i.e., severe burden (2), moderate burden (1), and No burden (0). In this study, a mean score of 1 is considered a moderate burden and 2 is considered a severe burden.

Caregiver distress was measured with depression, anxiety and stress scale (DAS-21).^19^ The depression anxiety and stress scale is a set of three self-report scales designed to measure the negative emotional states of depression, anxiety, and stress. The severity is represented as 0: did not apply to me at all, 1: applied to me to some degree, or some of the time, 2: applied to me to a considerable degree, or a good part of the time, and 3: applied to me very much, or most of the time. The scoring for depression: normal (0–9), mild (10–13), moderate (14–20), severe (21–27), extremely severe (28 above); anxiety: normal (0–7), mild (8–9), moderate (10–14), severe (15–19), extremely severe (20 above); and stress: normal (0–14), mild (15–18), moderate (19–25) severe (26–33), extremely severe (34 above).

Semi-structured interview schedule was used to collect the socio-demographic details of caregivers like age, gender, education, and occupation. The illness details such as symptoms, duration of illness, and severity of illness (Glasgow Coma Scale, GCS) scores were collected from patient files. GCS measures the severity of TBI. It can be scored as mild (13–15), moderate (9–12), and severe (8).

For continuous variables, mean, standard deviation; for categorical, nominal variables frequency, and the percentage were calculated. In addition, independent t test and Fishers exact were computed to see differences and associations for selected variables by using SPSS. Ethical permission was obtained from the institutional ethics committee. Oral and written informed consent were obtained before the recruitment of the participants in the study.

## RESULTS

Sixty primary caregivers participated in the study of which male caregivers were 75% and female caregivers were 25%. Caregiver's mean age was found to be 35.22±11.29 years. Majority of the caregivers belonged to Hindu religion (90%), educated up to high school (48.3%), married (75%), manual labors by occupation (46.7%), and hailed from a rural background (55%).

Traumatic brain injury survivors' mean age was found to be 40.12±19.45 years and gender distribution of the patients predominated in male (76.6%) as compared to female population (23.3%). Majority belonged to Hindu religion (91.7%), educated up to high school (51.7%), married (68.3%), and engaged in manual labor or daily wage (61.7%). Table 1 depicts the demographic characteristics of caregivers and TBI survivors.

The mean result of TBI survivors admitted in ICU were with severe injury (8.32±3.63), majority of them met with road traffic accident (RTA) (61.7%) under the influence of alcohol (30%), fall from height (25%), and assaulted (3.3%). Patients had received first aid treatment at the accident site (86.7%). Table 2 shows the illness details of the TBI survivors.

**Table 1:** Demographic characteristics of caregivers and TBI survivors

*Variables*	*Category*	*Caregivers* *n(%)*	*TBI survivors* *n(%)*
Gender	Male	45 (75)	46 (76.7)
	Female	15 (25)	14 (23.3)
Religion	Hindu	54 (90)	55 (91.7)
	Non-Hindu	6 (10)	5 (8.3)
Education	Illiterate	7 (11.7)	18 (30)
	High school (1–10)	29 (48.3)	31 (51.7)
	10+2 and Degree	24 (40)	11 (18.3)
Marital status	Married	45 (75)	41 (68.3)
	Unmarried	13 (21.7)	17 (28.3)
	Single	2 (3.3)	2 (3.3)
Occupation	Government	4 (6.7)	2 (3.3)
	Private	22 (36.7)	13 (21.7)
	Manual Labour	28 (46.7)	37 (61.7)
	Student	4 (6.7)	4 (6.7)
	Other Profession	2 (3.3)	4 (6.7)
Domicile	Urban	27 (45)	32 (53.3)
	Rural	33 (55)	28 (46.7)
Caregiver relationship	Spouse	9 (15)	-
	Daughter/son	17 (28.3)	
	Parents	24 (40)	
	Secondary relatives	6 (10)	
	siblings	4 (6.7)	

**Table 2:** Illness characteristics of TBI survivors

*Variable*	*n(%)*
Severe	40 (66.7)
Moderate	10 (16.7)
Mild or normal	10 (16.7)
RTA	37 (61.7)
Fall from height	15 (25)
Assault	2 (3.3)
Met accident under the influence of alcohol	18 (30)
First aid treatment received soon after accident	52 (86.7)

Mean scores depicted that caregivers experienced severe burden in the domains of financial burden (6.28±23.6), disruption of routine family activities (4.5±1.96), disruption in family leisure activity (3.25±1.57), and disruption in family interaction (1.86±1.58).

Further, psychological distress mean scores revealed that caregivers had experienced mild level of depression (12.15±4.84), moderate level of anxiety (12.85±5.20), and stress level found to be normal (13.5±4.22). Table 3 describes the descriptive statistics of caregiver burden and psychological distress of ICU caregivers.

Fisher's exact test depicted that severity of TBI injury was associated with caregiver burden. The severity of TBI was likely to increase the burden on caregivers at ICU. Table 4 shows the association between severity of injury (GCS score) and caregiver family burden.

Independent t test results showed that there were significant differences in caregiving burden between male and female caregivers at ICU (Male = 18.43±6.64, Female 14.29±4.83, t = 2.16, *p* <0.035). Mean scores showed male caregivers had experienced more caregiver burden compared to female caregivers. The result further showed there were significant differences between the level of psychological distress and caregiver family burden. Mean scores revealed caregiver who had experienced severe family burden were likely to experience severe depression (t = -4.35, *p* <0.001), anxiety (t = -2.80, *p* <0.007), and stress (t = -3.09, *p* <0.003). Table 5 shows the differences between psychological distress and caregiver family burden.

**Table 3:** Descriptive statistics of severity of TBI in patients, caregiver's burden and psychological distress

*Variable*	*Mean* *(N = 60)*	*Standard* *deviation*
Glass cow Come Scale (severity of injury)	8.32	3.63
Financial burden	6.28	2.36
Disruption of routine family activities	4.5	1.96
Disruption of family leisure	3.25	1.57
Disruption of family interaction	1.86	1.58
Effect on physical health of others	0.46	0.72
Effect on mental health of others	1.1	0.57
Depression	12.15	4.84
Anxiety	12.85	5.20
Stress	13.5	4.22

**Table 4:** Association between severity of injury (GCS score) and caregiver family burden

	*Family burden*				
*GCS*	*Moderate* *N(%)*	*Severe* *N(%)*	*Total* *N(%)*	*Fishers exact value*	*p value*
Mild	10 (83.3)	2 (16.7)	12 (100)	6.087	.050
Moderate	4 (40)	6 (60)	10 (100)		
Severe	17 (44.7)	21 (55.3)	38 (100)		

**Table 5:** Differences between caregiver's psychological distress, burden, and gender

*Variables*		*Mean*	*SD*	*t-value*	*p value* *p <0.05*
Depression	Mild/moderate	13.00	3.14	–4.35	0.001
	Severe	20.13	6.02		
Anxiety	Mild/moderate	12.38	4.21	–2.80	0.007
	Severe	18.70	6.13		
Stress	Mild/moderate	13.40	3.24	– 3.09	0.003
	Severe	19.71	6.23		
Burden	Male	18.43	6.64	2.16	0.035
	Female	14.29	4.83		

## DISCUSSION

Psychosocial and mental health concerns of caregivers in the area of TBI are less focussed and in particular, critical care is less studied in the Indian context. In this connection, our study is unique and contributes to the TBI research from a psychosocial perspective considerably.

The present study result showed that adults were more prone to RTA, and accidents took place under the influence of alcohol, fall from height, and assault. These are frequently reported as a cause of TBI in India.^20^ The result that indicated male were prone to the accidents compare to female and the severity of injury varied. Previous studies also reported that prevalence of road accidents are more among males compared to females.^21^ This finding is consistent with previous studies that many patients reach the tertiary care hospital with severe injury or vegetative state and require immediate ICU care.^22,23^ Irrespective of the degree of injury, TBI victims received first aid treatment before reaching ICU. This may be of good samaratarians and availability of emergency services nearby accident places. However, the quality of first aid, time taken to access immediate treatment, and utilization of prehospital care services may be explored in future studies.

Caregiver's demographic characteristics revealed that male caregivers took more caregiving responsibility than female caregivers. Spouses, daughter/son, parents, siblings, and secondary relatives had been involved in rendering day-to-day caregiving activities at ICU. The demographic characteristics of caregivers and TBI survivors are similar to previous studies conducted elsewhere in southern India.^7,16^

The study revealed that the severity of TBI injury was significantly associated with family caregiving burden in the domains of finance, disrupted family activities, family leisure time, and family interaction patterns. In addition, health and mental health of caregivers also affected considerably. In addition, caregiver experienced high levels of psychological distress in the process of caregiving at ICU. It was observed that caregivers who had experienced higher family burden were likely to experience higher psychological distress, i.e., depression, anxiety, and stress. Studies reported that moderate to severe TBI with persistent deficits leads to changes in the family system, and assuming family members to be a caregiver. Caregivers of individuals with TBI reported unhealthy family functioning leads to increased depression, higher levels of perceived burden, increased social isolation, poor work productivity, adjustment, and decreased psychosocial well-being.^15,24^ It was evident in the present study that male caregiver had experienced higher caregiver burden compared to female. This may be because of existing hospital rules that male patient must be accompanied by a male caregiver during inpatient care.

The study has few limitations in terms of study sample recruitment by using purposive sample and the majority of them were male caregivers. In addition, grief reactions were present but not measured in the crisis phase. Psychosocial interventions were not provided to address psychological distress. However, caregivers with depression were referred to a mental health professional for the evaluation and further management.

The study also observed that caregivers experienced higher family burden and severe psychological distress at ICU. Therefore, pre-operative counselling, family education, stress management, utilization of resources, and dealing with trauma reactions found to be effective in emergency and trauma care.^12^ The prior mentioned psychosocial interventions can be applicable to ICU caregivers as well. To conclude with, there is an immediate need to assess grief, psychological distress and burden of the caregivers at ICU, and provide psychosocial interventions for caregivers at ICU accordingly by trained professionals.
